# Exploring the Adenylation Domain Repertoire of Nonribosomal Peptide Synthetases Using an Ensemble of Sequence-Search Methods

**DOI:** 10.1371/journal.pone.0065926

**Published:** 2013-07-16

**Authors:** Guillermin Agüero-Chapin, Reinaldo Molina-Ruiz, Emanuel Maldonado, Gustavo de la Riva, Aminael Sánchez-Rodríguez, Vitor Vasconcelos, Agostinho Antunes

**Affiliations:** 1 CIMAR/CIIMAR, Centro Interdisciplinar de Investigação Marinha e Ambiental, Universidade do Porto, Porto, Portugal; 2 Molecular Simulation and Drug Design (CBQ), Universidad Central ¨Marta Abreü de Las Villas (UCLV), Santa Clara, Cuba; 3 Departamento de Biologia, Faculdade de Ciências, Universidade do Porto, Porto, Portugal; 4 Departamento de Biología, Instituto Tecnológico Superior de Irapuato (ITESI), Carretera Irapuato-Silao Km. 12.5, El Copal, Irapuato, Guanajuato, México; 5 CMPG, Department of Microbial and Molecular Systems, KU Leuven, Leuven, Belgium; The Centre for Research and Technology, Hellas, Greece

## Abstract

The introduction of two-dimension (2D) graphs and their numerical characterization for comparative analyses of DNA/RNA and protein sequences without the need of sequence alignments is an active yet recent research topic in bioinformatics. Here, we used a 2D artificial representation (four-color maps) with a simple numerical characterization through topological indices (TIs) to aid the discovering of remote homologous of Adenylation domains (A-domains) from the Nonribosomal Peptide Synthetases (NRPS) class in the proteome of the cyanobacteria *Microcystis aeruginosa*. Cyanobacteria are a rich source of structurally diverse oligopeptides that are predominantly synthesized by NPRS. Several A-domains share amino acid identities lower than 20 % being a possible source of remote homologous. Therefore, A-domains cannot be easily retrieved by BLASTp searches using a single template. To cope with the sequence diversity of the A-domains we have combined homology-search methods with an alignment-free tool that uses protein four-color-maps. **TI2BioP** (**T**opological **I**ndices **to** **BioP**olymers) *version 2.0*, available at http://ti2biop.sourceforge.net/ allowed the calculation of simple TIs from the protein sequences (four-color maps). Such TIs were used as input predictors for the statistical estimations required to build the alignment-free models. We concluded that the use of graphical/numerical approaches in cooperation with other sequence search methods, like multi-templates BLASTp and profile HMM, can give the most complete exploration of the repertoire of highly diverse protein families.

## Introduction

The Chemical Graph Theory (CGT) consists in the application of the graph theory to perform combinatorial and topological exploration of the chemical molecular structure. Currently, the CGT is being extended to bioinformatics through the introduction of two-dimensional (2D) graphs for comparative analyses of DNA/RNA and proteins without the use of sequence alignments. These 2D graphs or maps do not represent the “real structure” of the natural biopolymers but they have been very effective to inspect similarities/dissimilarities among them, either by direct visualization or by numerical characterization [1]. Examples of 2D artificial representations of DNA and protein sequences with potentialities in bioinformatics include the spectrum-like, star-like, cartesian-type and four-color maps [1]–[5]. These DNA/RNA and protein maps can generally unravel higher-order useful information contained beyond the primary structure, i.e. nucleotide/amino acid distribution into a 2D space. Their essence can be captured in a quantitative manner through numerical indices to easily compare a great number of sequences/maps [6]–[8]. One of the simplest numerical characterizations of sequences comprehends the use of topological indices. Topological Indices (TIs) are based on the connectivity between the elements composing the 2D graph in terms of whether they are connected or not [9], [10]. While several types of 2D maps have been developed for DNA/RNA and proteins, including their numerical characterization [11], the four-color maps application in bioinformatics has been mostly unexplored, being limited to illustrative examples on the comparative characterization of DNA and protein sequences [12]. However, the use of the four-color maps and its numerical characterization can cooperate with traditional homology search tools (e.g. BLAST, HMMs) to carry out an exhaustive exploration of functional signatures in highly diverse gene/protein families. Such exploration is effective when all family members are retrieved including remote homologs. Remotes homologues are divergent gene/protein sequences that have conserved the same biological function in different organisms. They can be harvest in the alignment algorithms twilight zone (<30% of amino acid identity) and have been traditionally detected by the use of more sensitive alignment-based methods like PSI-BLAST [13] and profiles Hidden Markov Models (HMM) [14]. The Nonribosomal Peptide Synthetases (NRPS) family can harbor remote homologous due to the high sequence divergence among its Adenylation domains (A-domains). In fact, all A-domain members cannot be retrieved easily by BLASTp using a single template [15]. NRPS are megasynthetases composed by several domains organized in clusters for the synthesis of oligopeptides with biological activities. A-domains are mandatory in each NRPS cluster being responsible for the amino acid selection and its covalent fixation on the phospho-pantethein arm as thioester, through AMP-derivative intermediate during the production of oligopeptides via non-ribosomal [16]. Cyanobacteria are a rich source of structurally diverse oligopeptides that are predominantly synthesized by NRPS. In *Microcystis*, a common cyanobacteria genus in eutrophic freshwaters, numerous bioactive peptides have been identified that can be mostly classified as aeruginosins, microginins, microcystins, cyanopeptolins, and anabaenopeptins [17]. In the present work we aim to annotate the A-domain repertoire in the proteome of *Microcystis aeruginosa* as a strategy to spot NRPS clusters. To handle the high sequence diversity of A-domains we used an ensemble of homology-search methods, including an alignment-free model that integrates the four-color-maps for proteins. **TI2BioP** (**T**opological **I**ndices **to** **BioP**olymers) *version 2.0*, available at http://ti2biop.sourceforge.net/ allows the calculation of TIs from the four-color maps for protein sequences [18]. Such TIs were used as input predictors for statistical techniques to build alignment-free models. We concluded that the use of an ensemble of sequence search methods (homology-based and alignment-free) can give the best exploration of the repertoire of highly diverse protein classes, such as the NRPS represented by its A-domains. The graphical method rendered a Decision Tree Model (DTM) that detected signatures of 22 A-domains in the proteome of *Microcystis aeruginosa* matching 19 out of 20 hits previously annotated as A-domains. The multiple-template BLASTp found exactly the 20 A-domain signatures annotated in the proteome, while the profile HMM detected the same 20 hits plus three additional ones. DTM and profile HMM identified, respectively, two and three A-domain signatures not found by multi-template BLASTp among the hypothetical proteins. The consensus detection of additional hits by the two sequence search methods provides clues for the presence of further A-domains remote homologues. The new A-domain variants found in the proteome of *Microcystis aeruginosa* could unravel the presence of novel NRPS clusters.

## Results

### Alignment-free model selection

**Figure 1:**
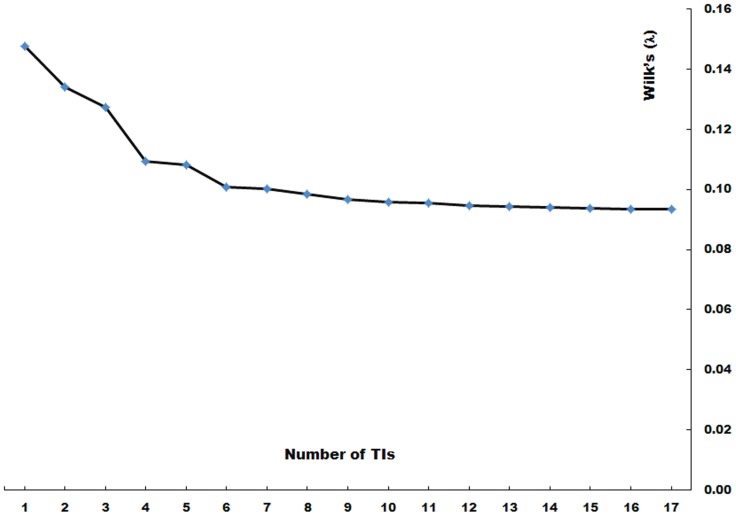
Assessing the relationship between the number of TIs entered in each model and the Wilk's (λ) values obtained for each one.

We computed 17 TIs that consist in spectral moment series (^fc^ **µ**_0_-^fc^ **µ**_16_) derived from four-color maps representing 8892 protein domains (138 A-domains and 8854 CATH domains) using **TI2BioP** (described in Methods and Database). The ^fc^ **µ**_0_-^fc^ **µ**_16_ series were used as input predictors to build classification linear models as the simplest relation between the response variable and the predictors. General Discrimination Analysis (GDA) best subset implemented in the *STATISTICA* software was used for such purposes [19]. We select the best subset of predictors that accounts for the more effective discrimination between A and CATH domains through plotting the λ variation against the number of predictors in the set of models. A parsimonious linear model was selected at the point where the λ start to decrease smoothly **(** **Figure 1** **).**

We found a linear classification function (**see equation below)** with four significant predictors (^fc^ **µ**_1,_^fc^ **µ**_2,_^fc^ **µ**_9,_^fc^ **µ**_12_) describing the topology of the four-color maps at short range (^fc^ **µ**_1,_^fc^ **µ**_2_) and at long range (^fc^ **µ**_9,_^fc^ **µ**_12_) interactions.(1)
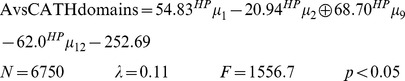
Where, *N* is the number of domain sequences used to train the classification model and the statistics parameters commonly used to evaluate linear functions (Wilk's statistical (λ) and Fisher ratio (F) with a probability of error (*p*-level)) [20], [21]. They provided values indicating a good power of discrimination (λ = 0.11) with significance (p(F)<0.05).

The model classification performance is shown in **Table 1** together with the classification results from other alignment-free models developed with non-linear techniques.

**Table 1:** Classification results for the three alignment-free models (GDA, DTM and ANN) in A-domains detection.

	Training					Test	
GDA	A-domain		CATH domain			A- domain	CATH domain
A-domain	**102**		0			**24**	0
CATH domain	7		**6641**			5	**2213**
Total	109		6641			29	2213
Sensitivity (Sv) (%)	93.58					82.76	
Specificity (Sp) (%)	100					100	
Accuracy (Acc) (%)	99.89					99.78	
F-score						0.99	
**10-fold CV**	**Sv**	**Sp**		**Acc**			
Average	93.58	100		99.89			

	Training						Test		
DTM	A-domain		CATH domain					A-domain	CATH domain
A-domain	**109**		0					**29**	0
CATH domain	0		**6641**					0	**2213**
Total	109		6641					29	2213
Sensitivity (%)	100							100	
Specificity (%)	100							100	
Accuracy (%)	100							100	
F-score								1.0	
**10-fold CV**	**Sv**	**Sp**		**Acc**					
Average	98.16	99.98		99.95					

	Training				Selection		Test	
ANN	A-domain		CATH domain		A-domain	CATH domain	A-domain	CATH domain
A-domain	**87**		0		**21**	0	**28**	0
CATH domain	0		**5313**		1	**1328**	1	**2213**
Total	87		5313		22	1328	29	2213
Sensitivity (%)	100				95.45		96.55	
Specificity (%)	100				100		100	
Accuracy (%)	100				99.92		99.95	
F-score							1.0	
**10-fold CV**	**Sv**	**Sp**		**Acc**				
Average	80.24	79.91		79.92				

**Figure 2:**
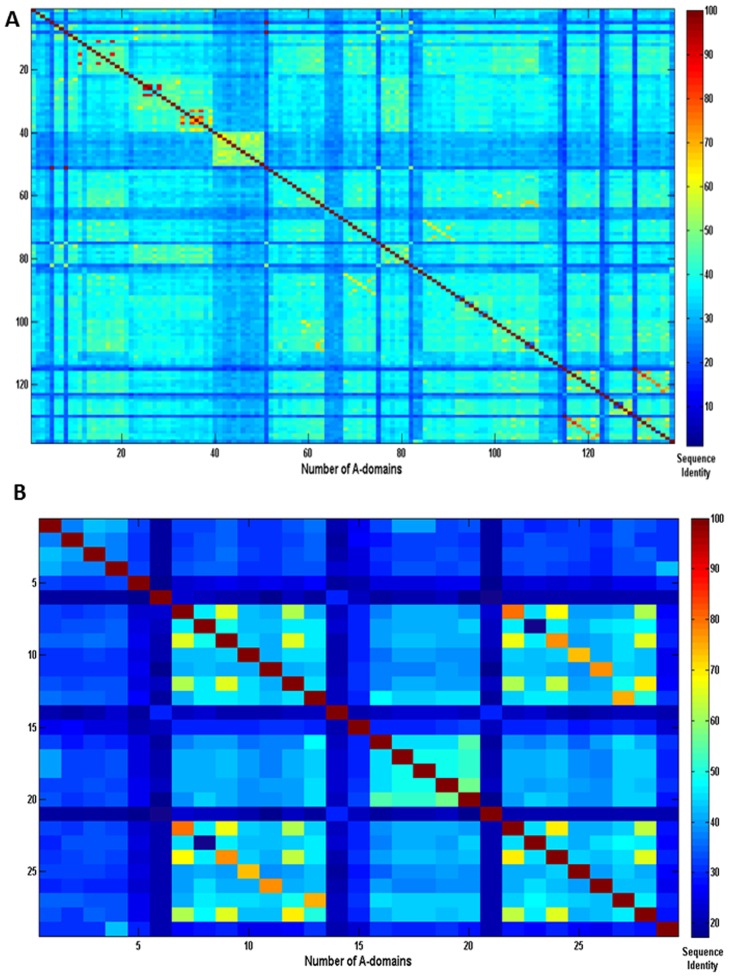
Dot plot for the global sequence identity matrix obtained by Needleman-Wunsch algorithm for A-domains. (A) All A-domains involved in the study. (B) A-domains of the test set.

GDA provides good classification results in detecting A-domains despite the members of this class ranged mostly between 10–40% of sequence identity **(** **Figure 2A** **)** and the CATH domains share less than 35% of sequence identity. Pair-wise identity is the most common cutoff used to decide the twilight zone for alignment algorithms [22]. Sequence alignments unambiguously distinguish between protein pairs of similar and non-similar functional and structural signals when the pairwise sequence identity is high (>40%). The signal gets blurred in the twilight zone of 20-35% sequence identity [22]–[24]. Particularly, the test set was made up of A-domains mostly sharing between 20 to 30% of amino acid identity **(** **Figure 2B** **)** and CATH domains with the diversity above-mentioned. Such test set matches into the twilight zone where generally remote homologous can be harvested.

The prediction power on the test set could be improved using non-linear models like Decision Tree Models (DTM) and Artificial Neural Networks (ANN) as can be seen below.

Although several alignment-free methods have been reported for improving classification accuracy in protein classes and super-families [25]–[27], DTM have been poorly explored to differentiate protein classes [28]. We used Classification Trees (CT) as an exploratory technique to obtain a DTM as predictive tools to detect A-domain signatures. The method found the ^fc^ **µ**_1_ and ^fc^ **µ**_2_ predictors as splitting variables to produce two decisions split at different values, respectively. The tree structure was very simple, two decision nodes (outlined in blue) and three terminal nodes (outlined in red) summing up a total of five nodes. The numbers of the nodes are labelled on its top-left corner and on the top-right corner are placed the label of the predicted class (A or CATH domain). The 6750 training sequences are assigned to the root node (first node) and tentatively classified as CATH domains or control set. CATH domains are chosen as the initial classification because they are numerically superior to A-domains.

The root node is split, forming two new nodes. The text below the root node describes the split. It indicates that protein sequences with ^fc^ **µ**_1_ values higher than or equal to 3817 are sent to node number 3 and tentatively classified as A-domains, by contrary domain sequences with ^fc^ **µ**_1_ values lesser than this value are assigned to node number 2 and classified in the control set (CATH domains). Similarly, node 3 is subsequently split taking the decision that sequences with ^fc^ **µ**_2_ values lesser than or equal to 11.12 are sent to node number 4 to be classified as A domains (109 cases). The remaining domain sequence with ^fc^ **µ**_2_ value greater than 11.12 are sent to node number 5 to be classified as CATH domains reaching 6641 cases well classified (100%).

**Figure 3:**
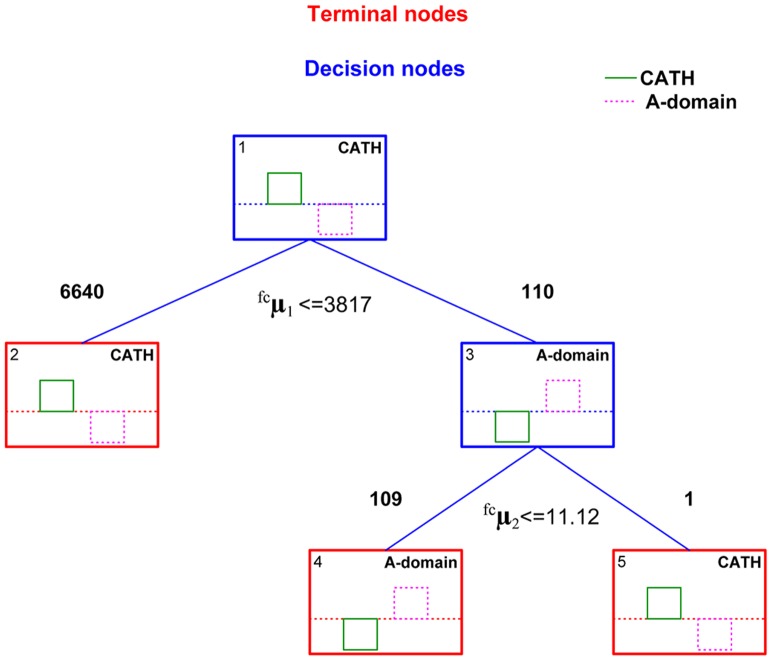
Architecture for the DTM. Decision Nodes are represented in blue and terminal nodes are in red. A-domains are labeled using an intermittent line. Otherwise CATH domains are signed by a continuous line. Labels at the right-corner of the nodes indicate tentative membership to A or CATH domain class. Numbers at the left-corner represent the node's number.

The tree graph presents all this information in a simple and straightforward way allowing processing the information easily. The histograms plotted within the tree's terminal nodes show the excellent performance of the DTM for the recognition of A- domain signatures **(** **Figure 3** **)**. The information from the tree plot is also available in **Table 2**.

**Table 2:** Tree structure in details, child nodes, observed class n's, predicted class, and split condition for each node.

Node	Left branch	Right branch	CATH	A-domain	Predicted class	Split constant	Split variable
1	2	3	6641	109	CATH	−3817.00	^fc^ **µ**_1_
**2**			**6640**	0	CATH	−11.13	^fc^ **µ**_2_
3	4	5	1	**109**	A-domain		
**4**			0	**109**	A-domain		
**5**			**1**	0	CATH		

Numbers in bold highlight the well-classified cases and the terminal nodes.

The classification results from the DTM development to recognize A-domain signatures on training and test sets are shown in **Table 1** as well as the results for the 10-fold CV procedure on the training set and the predictability on the test set. The classification improvement is remarkable in respect to the linear models.

ANN is one of the most popular non-linear modelling techniques in use today and has been frequently applied into bioinformatics [29]–[31]. The selection of input variables is a critical part of neural network design. We use the combination of our own experience and several feature selection algorithms (Forward, Backward and Genetic Algorithm Selection) based on Multilayer Perceptrons (MLP) available in the *STATISTICA Neural Networks* module for variable selection [19]. The ^fc^ **µ**_0_ and ^fc^ **µ**_1_ predictors were selected by consensus from the three methods. Then, a good starting point to set the topology of the MLP is to use one hidden layer, with the number of units equal to half the sum of the number of input and output units.

The **Table 3** shows the different MLP topologies used to select the right complexity of the ANN. The performance on training, selection and test progress were examined as well as its errors. The best model was the MLP profile highlighted in bold in **Table 3**, which showed the best accuracy on training, selection and test sets, minimizing its respective errors.

**Table 3:** Testing different topologies for the MLP on the A-domain classification using TIs from four-color maps.

Performance Summary for ANN							
	MLP Topologies	Train Accuracy	Selection Accuracy	Test Accuracy	Train Error	Select Error	Test Error
**1**	**MLP 2:2–1–1**∶**1**	**1.000**	**0.999**	**0.999**	**0.000**	**0.027**	**0.021**
**2**	MLP 2:2–2–1∶1	0.756	0.757	0.758	0.001	0.024	0.020
**3**	MLP 2:2–1–1–1:1	0.755	0.763	0.759	0.001	0.038	0.024
**4**	MLP 2:2–3–1∶1	0.756	0.755	0.760	0.016	0.033	0.035
**5**	MLP 2:2–1–2–1∶1	0.755	0.762	0.757	0.013	0.025	0.026
**6**	MLP 4:2–2–1–1∶1	0.756	0.757	0.759	0.006	0.022	0.020

Accuracy performance and error on training, selection and test sets.

The classification results derived from the best MLP profile to classify A-domains are shown in **Table 1**. This ANN-model also showed a higher accuracy level in classifying the training and test sets in respect to the linear model but a very similar performance in comparison to the DTM. However, according to the statistics from the 10-fold CV procedure carried out for each alignment-free model, the DTM shows the best statistics average **(** **Table 1** **)** being the most robust model reported among them. Therefore, DTM was the selected model to perform A-domains search among the proteome of *Microcystis aeruginosa.*

### Alignment-free approaches vs. homology-search methods in the detection of A-domains

We carried out a comparatively analysis to evaluate the sensitivity of other different alignment-free approaches and homology-search methods in respect to our graphical/numerical model to detect A-domains among the overall dataset (138 A-domains and 8 854 CATH domains) included in study. Such comparison was addressed to inspect the ability of our alignment-free approach to detect distant A-domains members (A-domains placed in the twilight zone) in the selected dataset. The Webserver PseAAC (http://www.csbio.sjtu.edu.cn/bioinf/PseAAC/) was used to generate alignment-free approaches based on amino acid composition (AAC) and pseudo amino acid composition (PseAAC) [32]. Both approaches provided classifiers to build up DTM under the same statistical parameters reported for our graphical/numerical-based model. Amino acids were weighted with their hydrophobicity values, similarly to the physicochemical property used for the four-color maps and λ values that reflect the sequence order effect was set to 0 if the AAC is only considered and 1 if we take into account the sequence order [33].

Most of the alignment-free classifiers have been based on AAC to predict protein cellular attributes and biological functions including remote homologs detection [26], [34]. One of the most popular alignment-free approaches is the Chou's concept of PseAAC that reflects the importance of the sequence order effect in addition to the AAC to improve the prediction quality to detect protein attributes [33], [35]. Classification trees were selected as the statistical technique to generate alignment-free models due to its simplicity and reliability to recognize the A-domain signature among the overall dataset **(** **Table 1** **)**.

On the other hand, homology-based searches for A-domains were performed by single-template BLASTp, multi-template BLASTp and profile HMM. These methods that show by definition different sensitivity to recognize distant homologs were evaluated considering their ability to retrieve all A-domains (close and distant members).

Our alignment-free model (DTM) generated by four-color maps outperformed alignment-free models (DTM) supported by AAC and PseAAC **(** **Table 4** **)**. Although A-domains share 10–40% of sequence identity with several members placed in the twilight zone, it was possible to retrieve all of them using four-color maps. In spite of the fact that the other two left alignment-free methods (AAC and PseAAC) showed lower sensitivity, they did not provide many false positives **(** **Table 5** **)**. It was also demonstrated the effect of the sequence order besides the AAC on the prediction quality; when λ was increased from 0 to 1, there was an improvement in all standard classification measures **(** **Table 4** **)**.

**Table 4:** Classification results for alignment-free DTM based on four-color maps, amino acid composition (AAC) and pseudo-amino acid composition (PseAAC) in the A-domains detection.

Four-color maps DTM	Training			Test
Sensitivity (Sv) (%)	100			100
Specificity (Sp) (%)	100			100
Accuracy (Acc) (%)	100			100
F-score				1.0
**10-fold CV**	**Sv**	**Sp**	**Acc**	
Average	98.16	99.98	99.95	

AAC (λ = 0) DTM	Training			Test
Sensitivity (%)	53.70			3.44
Specificity (%)	100			99.68
Accuracy (%)	99.25			98.44
F-score				0.07
**10-fold CV**	**Sv**	**Sp**	**Acc**	
Average	21.73	100	98.78	

PseAAC (λ = 1) DTM	Training			Test
Sensitivity (%)	67.89			20.68
Specificity (%)	99.80			99.77
Accuracy (%)	99.30			98.75
F-score				0.40
**10-fold CV**	**Sv**	**Sp**	**Acc**	
Average	21.73	100	98.78	

**Table 5:** True positives *vs*. false positives in the A-domain detection for different sequence-search methods among the overall dataset involved in the study.

Sequence-search method	True positive	False Positive
DTM (Four-color maps)	138	0
DTM (AAC)	59	7
DTM (PseAAC)	80	18
HMM (E-value = 10)	138	0
Multi-template BLASTp (E-value = 10)	138	0
BLASTp (E-value = 10)	138	6033
BlASTp (E-value = 0.05)	138	122
BLASTp (E-value = 0.01)	138	24
BLASTp (E-value = 0.001)	138	4
BLASTp (E-value = 0.0001)	138	0

Regarding homology-based methods sensitivity, classification results agreed with the fact that multi-template BLASTp and profile HMM are more sensitive than simple BLASTp. Both multi-template BLASTp and profile HMM easily retrieved all A-domain members at expectation values (E-value≤10) without reporting any false positive **(** **Table 5** **)**. However, the BLASTp search using a single template provided false positives (significant matches) among CATH domains at both high (E-value = 10) and relatively stringent cut-offs (E-values<0.05) **(Files S1–S5)**, which is considered statistically significant and useful for filtering easily identifiable homologs pairs [36], [37] (**Table 5**). False positives came up in simple BLASTp searches despite we had cleaned the negative set (CATH domains) from any A-domain signal (by the use of profile HMM-based searches). In contrast to multi-template BLASTp and profile HMM searches, the single-BLASTp search sensitivity did not show stability in identifying the A-domain signal among a benchmark dataset (CATH domains) when the classification parameter (E-value cut-off) was changed. Thus, due to the A-domain diversity, it is less reliable to extrapolate or apply BLASTp searches using a single A-domain template to an unknown test dataset such as an entire proteome. The multi-template BLAST reported by the PKS-NRPS developers was not only useful to detect A-domains with correct boundaries [15]; it also provided more sensitivity (no false positive) and reliability in the identification of this domain class from no stringent conditions (**Files S6**). In addition, both the profile HMM described in the methods section (**Files S7**) and the DTM built up from four-color maps profiles reached the top in classifying the positive and negative sets. These facts support that profile-based methods are more effective to deal with remote protein homology unless a muli-template BLASTp strategy or PSI-BLAST is conducted. The easy and reliable identification of A-domains by multi-template BLASTp, profile HMM and four-color maps in contrast to a simple BLASTp search and other alignment-free methods provided real clues about the ability of the four-color maps to identify A-domain members in the twilight zone given the evaluated dataset.

### An ensemble of methods to explore the repertoire of NRPS A-domains in Microcystis aeruginosa

The potentialities of the four-color maps and its numerical characterization to detect A-domains in the twilight zone are promising, as we showed previously. Detecting A-domains remote homologues with reliability in a proteome that contains a large diversity of proteins is a challenge for any sequence search method. As several homology-search methods have been assembled into a certain annotation resource to retrieve accurately all members from highly diverse gene/protein families [38], [39], we used our graphical alignment-free method not in competition but in cooperation with alignment procedures to explore the whole repertoire of A-domains, including the detection of new variants (remote homologous), in the proteome of *Microcystis aeruginosa.*

**Figure 4:**
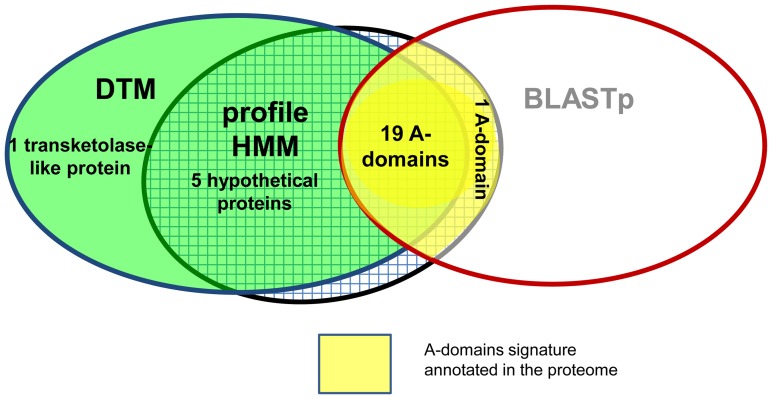
Re-annotation of the A-domains in the proteome of *Microcystis aeruginosa* by using an ensemble of algorithms.

The proteome of the *Microcystis aeruginosa* NIES-843 (http://genome.kazusa.or.jp/cyanobase) is encoded from a 5.8Mbp genome with 6 311 annotated genes; some of them codifying NRPS proteins as hybrids with polyketide synthases (PKS) representing a good target to evaluate the detection of A-domains. DTM was selected among the alignment-free models due to its excellent performance at low sequence identity and its simple way to recognize A-domains. We just calculate the TIs for a proteome and select A-domain signatures according to the DTM rule (^fc^ **µ**_1_≥3817 and ^fc^ **µ**_2_≤11.12) **(Files S8)**. DTM search detected 19 A-domain signatures that coincided with the previously annotation inferred for these genes in the proteome. Three additional cases were also detected as A-domains, but these cases have been previously predicted to be other protein signatures unrelated to NRPS A-domains in the proteome, namely a transketolase-like protein and the other two were hypothetical proteins. The putative hits with some remote relation to A-domains are probably found among the hypothetical proteins due to its unclear annotation. To increase the confidence and quality of the A-domains re-annotation, two sensitive homology-search methods were evaluated on the same proteome. We carried out multi-template BLASTp and profile HMM searches for A-domains in the proteome according to procedures described in the Methods section, respectively. Multi-template-BLASTp found 20 significant hits coinciding perfectly with the number of A-domains signatures in the annotated genome **(Files S9)**. The profile HMM detected 23 significant matches for the A-domain signature in the cyanobacteria proteome **(File S10)**. Twenty out of these 23 matches agreed with the multi-template BLASTp results and therefore with the current proteome annotation. The remaining three detected hits by the profile HMM were found among the hypothetical proteins, similarly to the alignment-free search **(** **Figure 4** **)**. These five hits retrieved by the use of two different sequence search methods among the hypothetical proteins could reveal the presence of additional A-domains remote homologues.

## Discussion

The potential usefulness of several graphical/numerical approaches to characterize genes and proteins for comparative analyses without the use of alignments has been recently reported by Randić *et al* [1], [40], [41]. We have extended this philosophy through the **TI2BioP** tool to characterize graphically and numerically large sequences databases [18]. The 2D Cartesian representation for genes and proteins and its simple numerical characterization were implemented in **TI2BioP** *version 1.0*, especially to deal with functional classification problems at low sequence similarity [8], [28], [42]. Our alignment-free models predictions based on graphical profiles have generally been used in cooperation with profile HMMs and experimental evidences [8], [28].

In this work we highlighted a practical utility of the four-color maps accompanied with sensitive alignment procedures to detect a functional signal among a highly diverse protein domains dataset including a proteome. The four-color maps construction was based on a similar procedure carried out to the building of 2D Cartesian maps for protein sequences, previously used with success to detect functional signatures at low homology level [28], [42].

Proteins four-color maps were modified by clustering the amino acids according to their physicochemical properties in four groups (polar, non-polar, acid and basic) labeled in the map with four colors. The numerical characterization of the four-color maps can describe homologous sequences (replacement between amino acids of similar properties) and remote homologous (important changes in the primary structure but still retaining the same biological function). While small changes in the sequence do not affect the topology of the map, this kind of amino acid substitution produces implicit numerical changes in the calculation of the TIs making possible the differentiation of the sequences. When an amino acid exchange occurs between different physicochemical groups of amino acids, this change affects the topology of the map and consequently affects significantly the TIs values estimation.

The TIs consist in the spectral moments series (^fc^ **µ**_0_-^fc^ **µ**_16_) describing the protein four-color maps. The topology of the protein four-color maps is determined by the sequence order and its amino acid composition (amino acid content according to the above-mentioned four groups). These two sequence features define the number and composition of the clusters formed in the map. The spectral moments series codify a range of information about the protein four-color maps that comprise the number of formed clusters in the map (^fc^ **µ**_0_) until the connectivity between the clusters in the map at different range (^fc^ **µ**_1_-^fc^ **µ**_16_). Our approach has a similar conceptual framework to the PseAAC introduced by Chou [33] but instead of using linear information (amino acid composition and sequence order) to get a vector representing the protein, four-color maps are built following similar rules but containing higher order information beyond the linearity of the sequence. Afterwards, the topology of such 2D graphs is described by node adjacency matrices used to calculate the spectral moments series as TIs.

The spectral moments series (^fc^ **µ**_0_-^fc^ **µ**_16_) were used to develop several alignment-free models with linear and non-linear statistical techniques. DTM and ANN showed a better performance in classifying A-domains in respect to linear models supporting that the identification of protein signatures are better assessed with non-linear models. DTM was the best-reported alignment-free model due to the reasons given in the previous section. Consequently, it was applied to get other alignment-free models based on AAC and PseAAC to inspect their sensitivity to retrieve all A-domains members. Such DTM displayed lower classification rates than those reached by the four-color maps based models **(** **Table 4** **)**. It seems that higher order patterns providing by the four-color maps are more effective in the detection of A-domains than linear sequence features driven by AAC and PseAAC. Therefore, the DTM based on four-color map patterns was selected to perform the alignment-free search for A-domains in the proteome of the cyanobacteria *Microcystis aeruginosa*.

Interestingly, DTM detected in the proteome two putative hits of A-domain signatures among the hypothetical proteins and later another three hypothetical proteins were detected as A-domains by the profile HMM **(** **Figure 4** **).** The sequence search methods based on profiles (graphical and alignment) were able to detect more hits than the 20 A-domains already annotated in the proteome, which were also detected by the multi-template BLASTp. Hypothetical proteins are greatly expanded in cyanobacteria and have been placed into the diversity of the nuclease superfamily by homology inference. Probably the graphical and HMM profiles detected signals of the A-domain signature among the diversity of the hypothetical proteins leading us to new variants of A-domains.

Both methods detected different additional hits as A-domains but they were found among the hypothetical proteins, which is a good clue for the presence of further A-domains remote homologues in the proteome of *Microcystis aeruginosa.* The use of an ensemble of methods provides more confidence to the predictions since each method exploits different features of the protein sequences. Four-color maps generate graphical patterns using the sequence order and the amino acid composition arranged into a 2D space. These graphical profiles are numerically described in a wide range of information by series of TIs, which characterize individually the sequences. Consequently, such TIs are flexible to be used for different classification problems (from high sequence identities until the twilight zone).

On the other hand, the profile HMM is based on amino acid positions conserved at low range through multiple sequence alignments in linear sequences. HMM profiles are proved sensitive tools for remote protein homology detection even when the sequence conservation is restricted to short motifs, as is the case of A-domains [16], [43].

The ensemble of the three sequence search algorithms (DTM, multi-template BLASTp and profile HMM) provided the best solution for the search of remote homologues among a highly diverse protein class.

## Methods

### Computational methods

**Figure 5:**
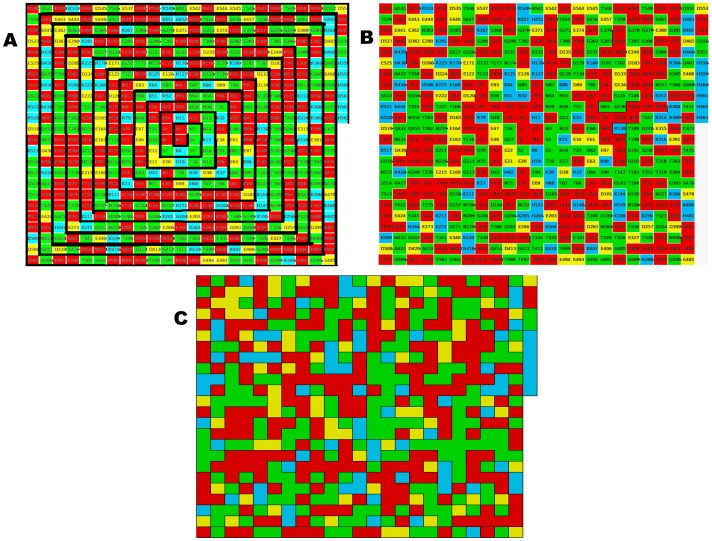
Steps for the four-color map construction of 1 pdb AMU. (A) Arranging the protein sequence into a square spiral. (B) Making up the clusters according to the amino acids properties: polar (green), non-polar (red), acid (yellow), basic (blue). (C) The final four-color map for pdb 1AMU.

**Figure 6:**
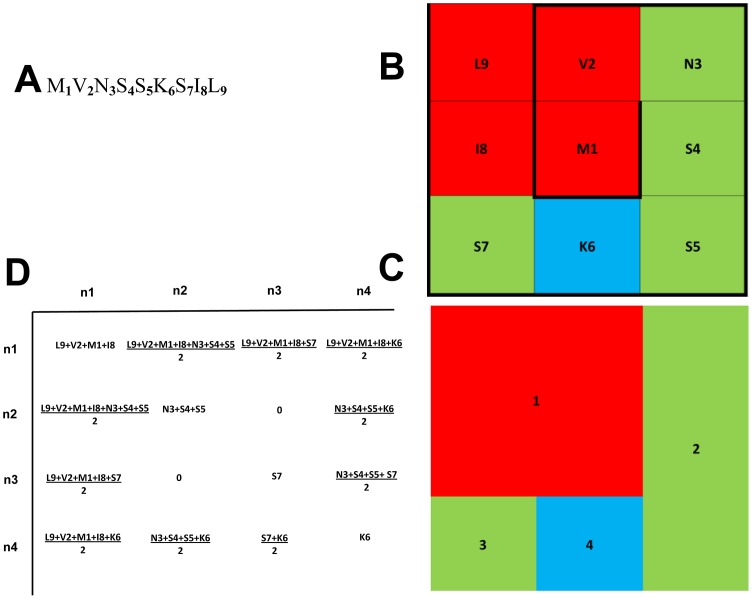
From the protein sequence to its numerical characterization. (A) The first nine aminoacids of pdb 1AMU. (B and C) Building the four-color map for A. (D) The definition of the node adjacency matrix derived from C the four-color map.

**TI2BioP** software *version 2.0* was used for the calculation of spectral moments as TIs associated with the protein four-color maps depicted below **(** **Figure 5** **).** Protein four-color maps are inspired on the Randic's DNA/RNA [44] and protein 2D graphical representations [12]; but instead of using the concept of virtual genetic code, we construct the spiral of square cells straightforward from the amino acid sequences. The four colors are assigned to the four amino acids classes (polar, non-polar, acid and basic) used previously by our group in Nandy's representation for proteins [28], [45]. A node adjacency matrix is defined to calculate the spectral moments to describe the topology of these proteins colored maps **(** **Figure 6** **)**.

**Figure 5** shows how the four-color map for the first A-domain structurally characterized is built up. It belongs to the Gramicidin Synthetase cluster isolated from *Brevibacillus brevis* (pdb 1AMU). Each of the four colors is associated with each one of the amino acid groups: polar (green), non-polar (red), acid (yellow), basic (blue).

### Database

#### Positive set

109 A-domain sequences from NRPS were collected from the major NRPS–PKS database (http://www.nii.res.in/nrps-pks.html) to conform the training set. The test set was made up of 29 A-domain sequences independently gathered from the subset of the NRPS-PKS hybrids (http://www.nii.res.in/nrps-pks.html). The sequence diversity among A-domains was explored comparatively using the Needleman-Wunsch (NW) algorithm.

#### Negative set

The starting group was made up for 8 871 protein sequences downloaded from the **CATH** (**C**lass, **A**rchitecture, **T**opology and **H**omology) domain database of protein structural families (version 3.2.0) (http://www.cathdb.info). We select the FASTA sequence database for all CATH domains sharing just the 35% of sequence similarity (<35% of sequence identity). The starting data was reduced to 8 854 CATH domains: 17 cases were removed because they showed the A-domain signature when an *hmmsearch* was performed against the AMP-binding profile HMM (PF00501). The members of the test set (2 213 sequences) were selected taking out at random the 20% from the 8 854 CATH domains; the rest 6641 CATH domains were used to train the models.

Each A-domain and CATH domain sequence retrieved was labeled respecting its original database ID code (**Files S11**).

### Numerical characterization of protein four-color maps through the spectral moments

The spectral moments are TIs calculated as the sum of the entries placed in the main diagonal of the bond adjacency matrix (**B)** between atoms for the small organic molecules. **B** is a square matrix of n x n row and column where its non-diagonal entries are ones or zeroes if the corresponding bonds or edges (n) share or not one atom. The different powers of **B** give the spectral moments of higher order to obtain the spectral moments series (**µ**_0_- **µ**_15_).
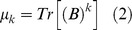
Where Tr is called the trace and indicates the sum of all the values in the main diagonal of the matrices ^k^ **B** = (**B**)^k^, which are the natural powers of **B** [46].

For the calculation of the spectral moments from the protein four-color maps, we consider each region of the map as a node made up for the amino acids clustering; two adjacent regions of the map sharing at least one edge (not a vertex) are connected. **B** is calculated in a similar way but instead of considering the adjacency relationships between bonds or edges, it is set between nodes. The number of nodes or clusters in the graph is equal to the number of rows and columns in **B**. Since a cluster is made up for several amino acids sharing similar physicochemical properties, the cluster is weighted with the sum of the individual properties (e.g. electrostatic charge (q)) of all amino acids placed in the cluster). The main diagonal of **B** was weighted with the average of the electrostatic charge (Q) between two adjacent clusters. The q values were taken from Amber 95 force field [47]. The calculation of the spectral moments up to the order k = 3 from the four colours maps is illustrated (downstream **figure 6** **)** using the first nine amino acids of pdb 1AMU (M**_1_**V**_2_**N**_3_**S**_4_**S**_5_**K**_6_**S**_7_**I**_8_**L**_9_**). The **figure 6** represents the four-color map built up for these nine amino acids, as well as its cluster adjacency matrix. q values are represented in the matrix as the amino acids symbols (M = 1.91, V = 2.24, N = 2.07, S = 2.09, K = 2.254, I = 2.02, L = 1.91).

Expansion of expression (2) for k = 0 gives the ^fc^µ_0_, for k = 1 the ^fc^µ_1_ and for k = 2 the ^fc^µ_2._ The node adjacency matrix derived from this 2D map is described for each case(2a)
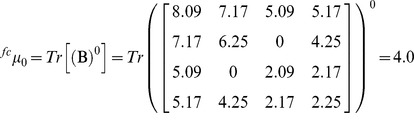

(2b)
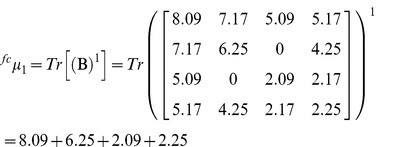

(2c)
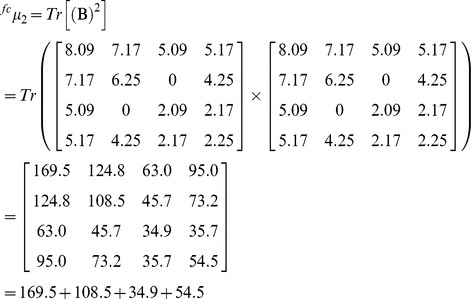



**TI2BioP** *version 2.0* arranges automatically all domain sequences (positive and negative sets) into four-colour maps and allows the calculation of spectral moments series (^fc^ **µ**_k_). **Files S12** shows the calculation of these indices to the positive and negative sets.

### Alignment-free models development with four-color maps TIs for A-domains detection

#### Linear models. General Discrimination Analysis

The General Discrimination Analysis (GDA) best subset was carried out for variable selection to build up the linear models [48]–[50]. All variable predictors were reviewed for finding the “best” possible sub model. The predictors were standardized in order to bring them onto the same scale. Subsequently, a standardized linear discriminant equation that allows comparison of their coefficients was obtained [51]. The model and variable selection was based on the revision of Wilk's (λ) statistic (λ = 0 perfect discrimination, being 0<λ<1). The Fisher ratio (F) was also inspected to indicate the contribution of one variable to the discrimination between groups with a probability of error (*p*-level) p(*F*)<0.05.

#### Non-linear methods. Decision Tree Models (DTM)

The development of the DTM was performed using the C&RT (Classification and Regression Trees)-style univariate split selection from the Classification Trees (CT) module of the STATISTICA 8.0 for Windows [19]. The C&RT examine all possible splits for each predictor variable at each node to find the split producing the largest improvement in goodness of fit. The prior probabilities were estimated for both groups with equal misclassification cost. The *Gini* index was used as a measure of goodness of fit and the ¨FACT-style direct stopping¨ was set to 0.1 as stopping rule to select the right-sized classification tree.

#### Artificial Neural Networks (ANN)

We used the Multilayer Layer Perceptron (MLP) network architecture as the most popular network architecture in use today. The selection of the subset of predictors that is most strongly related to the response variable was supported on the *Feature and Variable Selection* analysis of the ANN module from *STATISTICA* software [19]. The right complexity of the network was selected by testing different topologies to the MLP while checking the progress against a selection set to avoid over-fitting during the two-phase (back propagation/conjugate gradient descent) training algorithm. The selection set was randomly extracted (10%) from the training set. The test set was the same used for GDA and DTM representing an external subset (not used during training algorithms) to check the final network performance [52].

### Evaluation of models' performance and validation procedure

The performance of the all alignment-free models was evaluated by several statistical measures commonly used for classification: accuracy, sensitivity, specificity and F-score (it reaches its best value at 1 and worst score at 0). The robustness of the classification model was verified by a 10-fold cross-validation (CV) procedure on the training set. The CV statistics for each of the ten samples were averaged to give the 10-fold estimate for the accuracy, sensitivity and specificity [53]. In addition, a test set made up for 2242 domains was selected to evaluate the prediction power of each model.

### Ensemble of methods for re-annotation of A-domains NRPS in the proteome Microcystis aeruginosa

We used an ensemble of three methods for the re-annotation of the *Microcystis aeruginosa* proteome considering its repertoire of A-domains signatures.

1. The graphical method represented by the alignment-free model (DTM) to perform the A-domain search in the proteome. Spectral moments series from the four-color maps were calculated for the proteome of *Microcystis aeruginosa* NIES-843 (6 311 annotated genes) and later a simple rule was applied to detect A-domain signatures (^fc^ **µ**_1_≥3817 and ^fc^ **µ**_2_≤11.12).
2. A profile HMM for whole A-domain sequences was built as follows: (i) the 109 A-domain sequences used in training the alignment-free models were aligned by CLUSTALW [54], (ii) alignment was edited by Gblock software [55] to increase the alignment quality (iii), edited alignment was used as input for *hmmbuild* release 2.3.2 [14]. The generated profile HMM is used to search A-domains in the proteome of *Microcystis aeruginosa.*
3. The multiple-template BLASTp reported by the NRPS-PKS database developers for A-domain searches was used [15]. Multiple-template BLASTp consist in using each one of the 109 A-domains from the training set as template to evaluate each query of the proteome by BLASTp. BLOSUM62 scoring matrix, default values for gap penalties and E-value  = 10 were set as BLASTp parameters and just the best matches were retrieved.

## Conclusions

The utility of graphical approaches in bioinformatics has been demonstrated by the introduction of the four-color maps and the TIs as a cooperative tool for detecting remote homologous of A-domains in the proteome of *Microcystis aeruginosa*. Since each sequence search method extract different features from the protein sequences, their integration allow a more exhaustive description of certain protein class and therefore provide a higher yield for the detection of remote protein homologous. The knowledge of the complete repertoire of A-domains in the proteome of cyanobacteria species may allow unraveling new NRPS clusters for the discovery of novel natural products with important biological activities.

## Supporting Information

File S1 **BLASTp (E-value = 10) search for A-domains using a single template against the whole dataset.**
(TXT)

File S2 **BLASTp (E-value = 0.05) search for A-domains using a single template against the whole dataset.**
(TXT)

File S3 **BLASTp (E-value = 0.01) search for A-domains using a single template against the whole dataset.**
(TXT)

File S4 **BLASTp (E-value = 0.001) search for A-domains using a single template against the whole dataset.**
(TXT)

File S5 **BLASTp (E-value = 0.0001) search for A-domains using a single template against the whole dataset.**
(TXT)

File S6 **BLASTp (E-value = 10) search for A-domains using multiple templates against the whole data set.**
(XLS)

File S7 **Profile HMM (E-value = 10) search for A-domains against the whole data set.**
(TXT)

File S8 **Alignment-free search for A-domain signatures in the proteome of** ***Microcystis aeruginosa*** **.**
(XLS)

File S9 **Multi-template BLASTp search for A-domains in the proteome of** ***Microcystis aeruginosa*** **.**
(XLS)

File S10 **HMM profile search for A-domain signatures in the proteome of** ***Microcystis aeruginosa*** **.**
(TXT)

File S11 **Database used in the study. Fasta files for training and test series of A and CATH domains.**
(ZIP)

File S12 **IDs, training and prediction series, values of the TIs for A and CATH domains.**
(XLS)
